# The Influence of Sex and/or Gender on the Occurrence of Colorectal Cancer in the General Population in Developed Countries: A Scoping Review

**DOI:** 10.3389/ijph.2024.1606736

**Published:** 2024-04-10

**Authors:** Amalia Martinez, Pascale Grosclaude, Sébastien Lamy, Cyrille Delpierre

**Affiliations:** ^1^ Equity Research Team, Centre d’Epidémiologie et de Recherche en santé des POPulations, UMR 1295 (Équipe Labellisée Ligue Contre le Cancer), Inserm, University Toulouse III Paul Sabatier, Toulouse, France; ^2^ Institut Universitaire du Cancer de Toulouse-Oncopole (Institut Claudius Regaud), Toulouse, France; ^3^ Registre des Cancers du Tarn, Toulouse, France; ^4^ Equipe Labellisée Ligue Contre le Cancer, Toulouse, France

**Keywords:** gender, sex, incidence, colorectal cancer, nursing

## Abstract

**Objective:** Gender as the “sociocultural role of sex” is underrepresented in colorectal cancer incidence studies, potentially resulting in underestimated risk factors’ consequences and inequalities men/women. We aim to explore how literature focusing on differences between men and women in the incidence of colorectal cancer interprets these differences: through sex- or gender-related mechanisms, or both?

**Methods:** We conducted a scoping review using PubMed and Google Scholar. We categorized studies based on their definitions of sex and/or gender variables.

**Results:** We reviewed 99 studies, with 7 articles included in the analysis. All observed differences between men and women. Six articles examined colorectal cancer incidence by gender, but only 2 used the term “gender” to define exposure. One article defined its “sex” exposure variable as gender-related mechanisms, and two articles used “sex” and “gender” interchangeably to explain these inequalities. Gender mechanisms frequently manifest through health behaviors.

**Conclusion:** Our results underscore the need for an explicit conceptual framework to disentangle sex and/or gender mechanisms in colorectal cancer incidence. Such understanding would contribute to the reduction and prevention of social health inequalities.

## Introduction

The impact of sex and gender on health is a key topic in health research. Sex involves anatomy, physiology, genes and hormones, assigning male or female sex from birth [1].

Gender is a more recently studied concept in the field of health research, based on a 1970 definition: “gender refers to the social differences observed, experienced, prescribed or favoured, based on the sex assigned at birth.” Gender, rooted in social and political contexts, dictates “feminine” and “masculine” roles, impacting behaviours, expectations, and labor divisions. Gender is therefore “the socio-cultural role of sex,” linked to social and economic status, and thus acts as a social determinant of health and social inequalities in health [2, 3].

Colorectal cancer (CRC: ICD-11 codes: 2B90 – 2B9) is the third most common cancer and the second leading cause of cancer deaths worldwide in 2020 [4]. Diagnosis at an early stage enables rapid treatment and improves patient survival. In developed countries, there has been a reduction in mortality [5, 6]. Inequalities in the incidence of CRC have been identified in relation to ethnic origin, socio-economic position and other socio-demographic factors [7, 8]. Gender differences have also been highlighted in terms of management [9–11], survival [12, 13] and screening [14, 15]. Although gender differences in the incidence of CRC have been highlighted by the major epidemiological data sources [16, 17], few studies have explored the underlying mechanisms. However, several commissions and policies [18, 19] reaffirm the importance of conducting research that integrates both gender and sex. The most recent one, “Women, power, and cancer: a Lancet Commission,” [20] underscores the urgency of considering gender as a key determinant of health inequalities in cancer research. Understanding the distinction between sex and gender definitions is essential for analyzing social inequalities in colorectal cancer incidence from a sex and gender perspective. In this way, it will be possible to a better understanding of the origins of the differences in risk of colorectal cancer between men and women.

For this reason, we propose to conduct a review of the literature focusing on the differences between men and women in incident cases of CRC. More specifically, our aim is to carry out a scoping review in order to understand how these articles interpret sex and/or gender differences and to identify the mechanisms associated with these differences.

## Methods

We conducted a scoping review following the methods recommended by Arksey and O’Malley [21]: 1) identification of our research question; 2) identification of relevant studies; 3) selection of studies; 4) extraction of important data; 5) synthesis of results. Our study complies with the PRISMA-ScR statement (Reporting Items for Systematic Reviews and Meta-Analyzes extension for Scoping Reviews) [22].

In this study, we use the definition of gender expressed by C.L. Ridgeway and S.J. Correll, which presents a consensus with the many definitions found. Gender is therefore defined as “institutionalized system of social practices for constituting people as two significantly different categories, men and women, and organizing social relations of inequality on the basis of that difference” [23].

1) Our research question was to investigate sex and/or gender differences in the incidence of colorectal cancer in developed countries (countries with a high human development index) over the past 20 years.
2) Systematic searches were undertaken from April 2022 to August 2022, using the MEDLINE (PubMed) and Google Scholar databases. Keywords used in the search included MeSH database proper terms for Medline. We used Mesh term search, to identify terms used by scientific to define “sex”, “gender” and “colorectal cancer”. Subsequently, we used these terms directly in the Pubmed database and free-form terms relating to “sex” AND/OR “gender” AND “colorectal cancer” AND/OR “colon cancer” AND/OR “rectal cancer” present in article titles for the Google Scholar database. The advanced search in Google Scholar does not allow a keyword search, so we carried out a title search. To guarantee our results found in the two databases, we also carried out a second search on PubMed by article title. However, we kept the first search equation by keyword, which gave us more baseline articles: 26 texts compared with 21 with a search by title. The search equations used are shown in. In addition, a manual selection of article references was carried out.
3) Selection of articles

The inclusion criteria for the articles were as follows

*Type of article*: peer-reviewed articles and empirical articles (controlled trials, cohort or cross-sectional study designs).

*Explanatory variable*: sex and/or gender. Articles where the cited objective was the study of sex and/or gender (noted sex/gender) as an explanatory variable and/or risk factor for colorectal cancer.

*Variable of interest*: incidence of colorectal cancer.

*Date of publication*: 2000–2022. These time limits were chosen because, since the 2000s, European countries and the United States have laid out concerted programs to act against colorectal cancer on the basis of convincing epidemiological data. In 1995, the U.S. Preventive Services Task Force (USPSTF) drew up their Guide to Clinical Preventive Services, enabling the federated societies of gastroenterology and hepatology to lobby for the inclusion of colorectal cancer screening in Medicare benefits [24]. In 2000, the European Commission’s Cancer Experts Group recommended the introduction of colorectal cancer screening [25]. In addition, numerous studies have demonstrated that CRC mortality reduction was possible by introducing screening for occult blood in the stools, which led to the inclusion of this screening in the European Code against Cancer [26–28]. Furthermore, an awareness was born in the 1990s in the United States, around integrating the gender dimension in medicine and research. The government structure for public health research, the National Health Institute (NIH), adapted its research policy to fairly consider the question of sex and gender in health research. From the 2000s, Europe became aware of the importance of health research based on sex and gender equity. Gendered medicine was first set up in Germany and Northern Europe. The reflection on the integration of these notions in public health is still evolving since in France, in 2013, the INSERM ethics committee developed a working group on the theme “gender and health research” to raise researchers’ awareness of health inequalities related to sex and gender. This period ensured a degree of uniformity in the healthcare environment, closely linked to the countries’ desire for economic and health development. Additionally, these countries adopted a desire to reduce health inequalities related to sex and gender during the same period [29].

*Studies conducted in developed countries*: The relationship between a country’s economic development and the health status of its population has already been well established in numerous studies [30, 31]. In order to ensure consistency in lifestyles, levels of social development and life expectancy, we concentrated on studies conducted in developed countries. As defined by the United Nations Development Programme (UNDP), these countries had to have a Human Development Index greater than or equal to 0.8 and belong to the Organisation for Economic Co-operation and Development (OECD), Eastern Europe, Central Europe or the Commonwealth of Independent States (CIS) [32]. As their populations have a higher life expectancy [33] and access to a better healthcare system than developing countries, the prevalence of CRC is higher [6]. In addition, most developed countries have adopted a similar lifestyle, with some lifestyle practices becoming risk factors for colorectal cancer [33–35].

*Language*: French and English.

*Population*: general population, adults diagnosed with incident colorectal cancer.

The exclusion criteria for articles were as follows

Colorectal cancer studies outside the incidence phase; non-article formats such as citations, posters and commentaries.

We used the PRISMA protocol to draw up our criteria grid for selecting articles [22]. After eliminating duplicates, the selection was carried out in three stages: 1- reading the titles, which had to relate to the research question and include the words in the search equation, i.e., include the terms or synonyms for: colorectal cancer, incidence, sex or gender or men and women, 2- reading the abstracts with particular attention to the objective of the study, and 3- reading the articles in full. This was done by a first researcher (AM), then half of the articles selected were given for a second reading to a researcher (CD), and the other half to another researcher (SL). In the event of disagreement over a selection of articles, a third reading was carried out by the researcher who had not read the article in the previous stages (CD or SL).

4) The extraction of the data

The extraction of the data collected included the identification of the articles (name of the first author, year of publication, title of the publication); the design of the study; the population included; the definition of “sex” and/or “gender”; the nature and location of the cancer; the description of the influence of sex/gender on the risk of cancer and the description of the adjustment variables linked to the association of sex/gender on CRC.

5) Summary of results

We described the selected studies with particular attention to their use of sex and/or gender as an expression of socio-cultural role (gender) or as a biological attribute (sex). We classified the studies according to the approach used by the authors to characterise the sex and/or gender variable. This qualitative classification was based on the mechanism explained by the authors to explain the effect of the sex/gender variable on the incidence of CRC. If the authors expressed a social mechanism explaining the differences between women and men, for example, the level of education, or different behaviours according to sex, we classified the article as gender-related if not, we classified it as referring to sex.

## Results

### Selection of Articles

**FIGURE 1:**
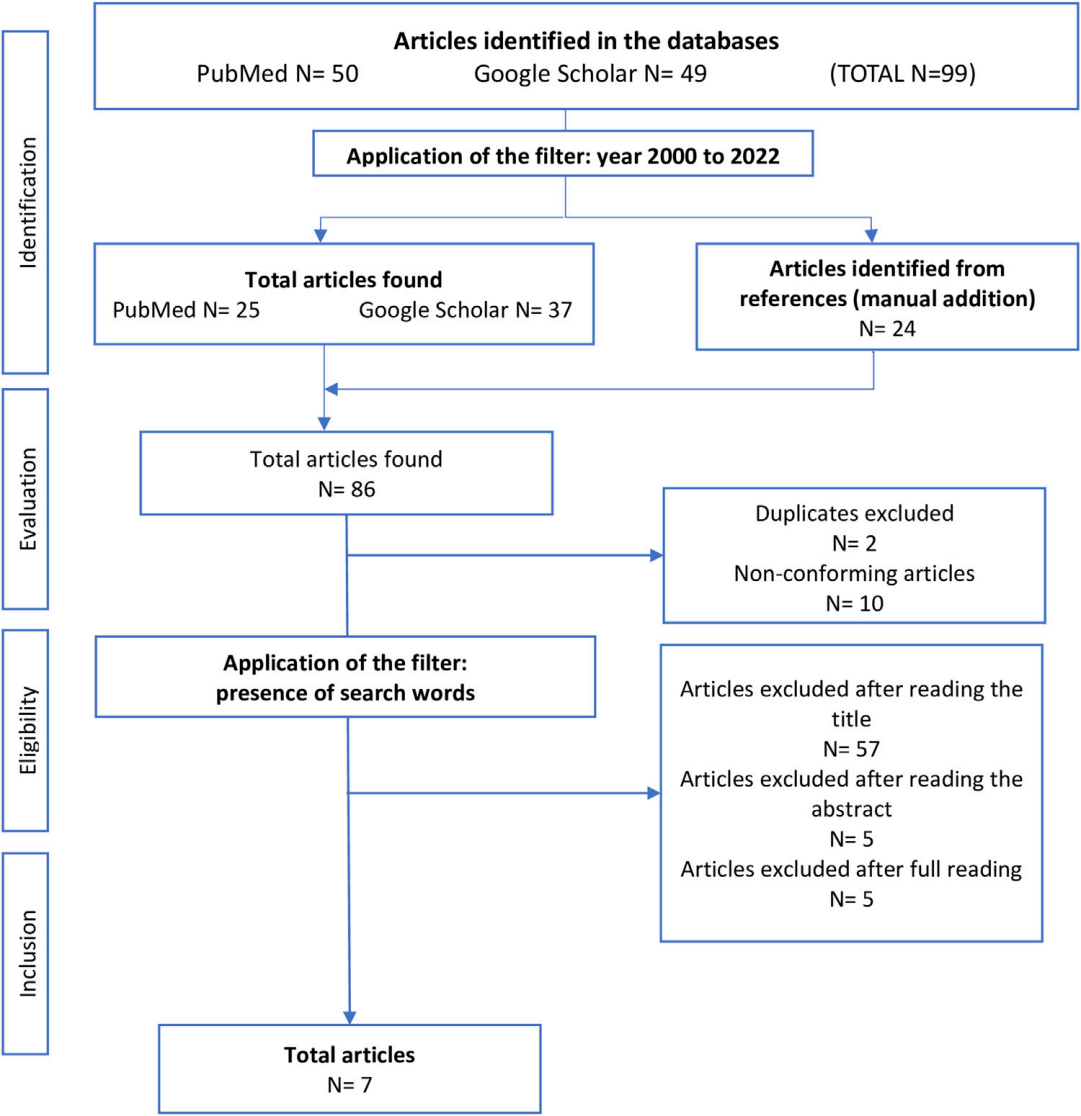
Selection flowchart (Toulouse, France, 2023).

A total of 99 articles were found in the two search databases: 50 articles in PubMed and 49 in Google Scholar. After applying the years filter (from 2000 to 2022), 62 published articles were retained, to which 24 articles were manually added from their references. After applying the exclusion criteria and removing duplicates, 74 articles were retained. Firstly, we selected based on title and eliminate the articles that did not contain the keywords in the search equation (*n* = 57) (Supplementary Appendix). Then, after reading the abstract, five articles were excluded, and after reading the full text, four review articles and one article dealing with the influence of the interaction between gender and Body Mass Index (BMI) on the risk were deleted. The final sample therefore comprised seven articles (Figure 1).

### Description of Selected Articles

Four of the selected studies were published between 2009 and 2011, only 1 year apart, including 2 in 2011 [36–39]. Of the seven studies selected, five were conducted in the United States [36, 38–41], one in Germany [37] and one in the United Kingdom [42].

With regard to the data used, all the studies described the general population, six of them via registries: three studies used exclusively data from SEER (Surveillance, Epidemiology and End Results) covering regions of the United States [36, 38, 39]; one study data from The North American Association of Central Cancer Registries (NAACCR) (including the SEER registry and The North American Association of Central Cancer Registries (NAACCR)) [40]; one study used data from US Cancer Statistics [including the SEER registry and the National Program of Cancer Registries (NPCR)] [41] and the last used data published by UK cancer registries [42]. Finally, the only study not using registry data collected its data from a statewide cohort study in Germany in 2005, which included patients who had undergone a colonoscopy for the first time the results of which results confirmed the presence of colorectal cancer [37].

In terms of inclusion periods, one study covered a population included between 1992 and 1997 [40], followed by successive inclusion periods from 2004 to 2018 for the most recent study (2021) [41]. Only three studies included people of all ages [38, 42], the other four having established inclusion criteria linked to specific age groups: Petrick et al. were interested in people aged between 20 and 74 years; Cheng et al. focused on people aged between 30 and 85 years plus; and Abotchie et al. and Hoffmeister et al. set their age limits at 40 years or more and 55 years or more, respectively.

#### Use of Sex and/or Gender in the Articles

Among the seven studies, the word “sex” appeared 59 times in the article (abstract, introduction, method and results) and 54 times the world “gender”. Only two studies used the word gender in their title, in the study by [39] published in 2011 and in the study by [40], which is the oldest of the studies analysed, i.e., 2001. The [39] study cited the word “gender” the most times (29 times). These two articles did not mention the word “sex” once.

Among the five other articles, three articles ([36], [38], [41]) did not mention the word “gender” at all, and two articles which used both the terms “sex” and “gender.” The [36] article reported the word “sex” the most times (19 times). Of the five articles using the word “sex”, only one provides a definition. According to [36] “Sex, that is, being male or female [.]. Differences in health and illness are influenced by individual genetic and physiological constitutions, as well as by an individual’s interaction with environmental and experimental factors.” [36] have defined an exposure variable called “sex” which also includes gender mechanisms since, according to them, sex exposes individuals to different cancer risk factors, such as biology but also exposure to carcinogenic substances and different risk behaviours (Supplementary Table S3).

#### Mechanism of Gender

Despite the absence of a clear explanation, six articles express gender mechanisms associated with their exposure variable, two of which name it “sex” [36, 38], 2 “gender” [39, 40] and 2 both “sex” and “gender” [37, 42]. The authors of these six articles put forward the hypothesis that men and women have different social experiences which expose them unequally to CRC risk factors [36–40, 42], making it possible to interpret their exposure variable as gender-related. The underlying social mechanisms used by the authors to highlight inequalities between men and women in the incidence of CRC correspond for the most part to health behaviours linked to smoking status and use of the healthcare system [37, 38, 40, 42]. Abotchie et al. [39] are the only authors to analyse gender disparities in CRC incidence rates by stratifying by geographical area. This makes it possible to highlight the existence of potential etiological factors linked to gender, such as the standard of living and access to the healthcare system in the geographical area under consideration.

Only one article [41] analyses the “sex” exposure variable as relating to sex assigned at birth. The authors use the risk factors for CRC established by the American Cancer Society, in particular alcohol, physical inactivity, obesity and diet, but hypothesise that the differences between men and women in the incidence of CRC are linked to undiscovered biological and genetic factors (Supplementary Table S3).

## Discussion

### Main Results

Our first objective was to identify the number of articles exploring the differences between men and women in terms of new cases of colorectal cancer. We identified seven articles published between 2000 and 2022 studying the relationship between sex and/or gender and the incidence of CRC, which shows that few studies focus specifically on this difference. Most of the studies focusing on this difference are mainly based on American data [36, 38–41]. This result indicates that there are still too few studies on the subject of inequalities in the incidence of CRC between men and women, particularly in non-American contexts.

Our second objective was to describe how the sex and/or gender exposure variable is defined. Among the seven studies, the definitions were implicit or imprecise, making it difficult to understand how the variable was used and whether or not a gender mechanism was involved. Only one article clarified the definition of its “sex” exposure variable [36], and the definition provided included dimensions of both biological sex and gender. Furthermore, among the studies using gender mechanisms, two studies named their exposure variable both “sex” and “gender” and two studies named it “sex”. Our work therefore highlights confusion and substitution of the terms “sex” and “gender”.

In the end, only one of the 7 studies included in our study analysed its exposure variable as “sex assigned at birth”. The other 6 studies highlighted gender mechanisms associated with health behaviours, in particular smoking and use of healthcare.

After analysing the work of these seven articles, we hypothesize that researchers in the field of CRC have a poor understanding of the definitions of the terms sex and gender. In addition, these terms are often used synonymously or interchangeably in these articles and in scientific articles in general [43–45].

### Strengths and Limitations of the Articles

The countries where the seven selected studies were conducted were the United Kingdom, the United States and Germany. These articles report on public health problems in a small range of developed countries where it is possible to integrate data infrastructures such as registers and administrative data. This systematic population-based data collection ensures that the populations most affected by CRC are representative. In addition, registry data provides complete and continuous information on incident cases of CRC.

The disadvantage of using registry databases is the lack of information on environmental and social variables. Data on individuals’ eating habits, physical activity, alcohol and tobacco consumption, and obesity or overweight are necessary in analyses of the influence of gender on the risk of CRC, as these are recognised risk factors which are unequal according to sex [34, 46, 47]. In this respect, only the study by [37], by merging national data with data obtained via a standardised questionnaire, makes it possible to add socio-demographic and lifestyle factors. They adjust for CRC risk factors such as Body Mass Index (BMI), consumption of red meat and alcohol, physical activity, and personal and family history. They also include the level of education, which provides information on the socio-economic position of individuals. Socioeconomic position is made up of various socioeconomic factors such as level of education, income and socio-professional category, which may reveal gender mechanisms because they are likely to influence the relationship between sex/gender and the incidence of CRC. Indeed, European and Finnish studies [48–50] show that the incidence of CRC was higher in people with a high level of education and in advantaged socioeconomic groups than in those with a low level of education and in disadvantaged socioeconomic groups. In addition, two Finnish studies show that differences in CRC incidence associated with education and socioeconomic group were more favourable in men than in women [48, 51]. However, it seems that populations with a low socio-economic position are more exposed to the aetiological factors of CRC [52–54]. Moreover, in their systematic review on inequalities in colorectal cancer screening participation, [55] observed a lower participation rate among population with a low socioeconomic position. They also highlighted higher CRC screening participation among women without a corresponding overincidence of CRC in this group. In contrast, men participate less in screening but have a higher incidence of CRC. This, suggest the existence of gender inequalities stem from in health-seeking behaviors by gender, as well as more frequent exposure to risk factors by gender. However, only a small fraction of the studies reviewed examined screening participation from a gender perspective (only 2 on 87), which is consistent with our results.

Furthermore, differential environmental exposures, including occupational exposures, could potentially influence CRC incidence. The International Agency for Research on Cancer (IARC) has categorized agents by organ type, some of which may stem from occupational exposures and cause cancers of digestive tract [56]. This link has been confirmed by some studies for specific occupational exposure to silica [57] and specific professions such as firefighters [58], veterinarians until 1990 [59], and paper mill workers [60]. Nevertheless, interpreting the relation between occupational exposure and cancer incidence in these studies remains challenging due to the lack of integration of associated risk factors in the analyses. This could explain the lack of evidence for specific risk factors for CRC.

It remains to be seen if these indicators of socioeconomic inequalities can be considered in the relationship between sex and the incidence of CRC from a gender perspective. However, no study has incorporated these factors as mediators of the relationship between sex and the incidence of CRC. Despite the inclusion of socioeconomic factors in their analyses, [37] controlled for the effects of sex on the incidence of CRC but did not identify any gender mechanisms.

The use of registries data alone makes it possible to obtain data without selection bias, but limits the possibility of highlighting gender mechanisms due to the lack of variety in social and behavioural data. Nevertheless, it is possible to obtain information on the spatial environment of individuals via registries. Indeed, the study by [39] included geographical area in their analyses, which is a strength in terms of the underlying aetiological factors linked to gender. By stratifying by geographical area, inequalities between men and women in terms of CRC incidence can be revealed, potentially linked to environmental exposures and different standards of living and lifestyles. Furthermore, it has been shown in the literature that the socioeconomic deprivation index of regions influences CRC incidence rates [61–66], which accentuates the importance of including data on the living environment of individuals. [67] support the importance of taking account of these social, behavioural and spatial factors as gender mechanisms. In fact, they highlight the influence of the social and physical environment in which an individual interacts, integrated differently between men and women, in the relationship between the individual and a state of health. As a result, the biological differences observed in individuals of different sexes may derive from gender mechanisms, through experience of their environment. Thus, the inclusion of the living environment from a geographical and social point of view in the relationship between men/women and the incidence of CRC is interesting to explore.

The seven articles selected note differences in the incidence of CRC between men and women, attributed to differences in sex assigned at birth and gender. However, the analyses do not explore sufficiently the socio-demographic and behavioural aetiological factors to methodologically integrate the gender dimension.

### Perspectives

In order to move towards a clearer, more comprehensible science that reflects exactly what researchers want to put forward, it is necessary to specify whether, when and how gender mechanisms, sex-related biological characteristics, both, or neither, have an influence on CRC risk. A number of studies have taken up this challenge and made it possible to operationalise methods for exploring the mechanisms explaining the differences in health between men and women [1, 68]. The work of [68] provides a solid basis for attempting to measure the diversity of gender and sex mechanisms and thus understand how they interact to produce a state of health. Nevertheless, gender mechanisms are numerous and difficult to grasp. Based on the conceptual analysis model explained by Colineaux et al., we propose a conceptual framework (see Supplementary Figure S2) to help take better account of gender mechanisms via mediating factors (health literacy, socioeconomic position, risk behaviour, etc.) in the incidence of colorectal cancer. This conceptual framework allows us to hypothesise about potential gender mechanisms influencing CRC incidence. We assume that our exposure variable, sex at birth, is associated with “gendered” social factors, which lead to potentially risky behaviour and condition health literacy levels in adolescence and adulthood. We hypothesise that the influence of gender on the incidence of CRC is mediated by these factors, which are coded and implemented differently according to gender. We call this link the gender mechanism. Of course, confounding factors can have an impact on these relationships, which is why it is important to control for their effects. For example, an unfavourable socio-economic position of the parents will influence the living environment in childhood, which may introduce unequal risk behaviours between men and women in adulthood. Our hypothesis is that all these factors determine the onset of CRC in adulthood and that the explanation of gender mechanisms requires considering sex at birth and mediating factors influenced by sex, which in turn influence the incidence of CRC.

### Strengths and Limitations of Our Scoping Review

In order to meet our research objective, we limited our literature review to peer-reviewed articles published in English, without considering the “grey” literature. Furthermore, we chose to focus articles containing the keywords from our search equation only in their titles and which use sex and/or gender as explanatory variable. This focus could bias our selection of articles. However, this analysis was not intended to be systematic, but to provide an overall picture of the influence of gender on the incidence of CRC in the scientific literature. The literature on the subject is poor, making it unclear whether there are differences between men and women in the incidence of CRC and whether these differences are the result of gender and/or sex mechanisms. The paucity of research available on the subject highlights the need to clarify, validate and standardise definitions of sex and gender, as well as strategies for taking gender into account. For all these reasons, a scoping review approach seems appropriate and relevant.

### Conclusion

Little work has been done to analyse the influence of gender/sex on the incidence of colorectal cancer. The seven articles analysed use registry data, limiting the ability to highlight gender mechanisms due to the lack of available social and behavioural data. This work underscores the need for further research on the differences in CRC incidence between men and women, clarifying the conceptual framework used to explain these differences.

### Precis

The few studies that have examined differences in colorectal cancer incidence related to sex and/or gender demonstrate a lack of definitions and significant confusion surrounding the terms of sex and gender, as well as their associated mechanisms. Our findings emphasize the need to explicitly define the theoretical framework and underlying assumptions.
