# Supplementary material for: The Influence of Sex and/or Gender on the Occurrence of Colorectal Cancer in the General Population in Developed Countries: A Scoping Review
Source: Int J Public Health. 2024 Apr 10;69:1606736. doi: 10.3389/ijph.2024.1606736 (PMC11039791; doi:10.3389/ijph.2024.1606736)
Supplement: Supplementary file 1 [file Table1.pdf]

Table S1: Search equations in the various databases (Toulouse, France. 2023)

Databases	Research equations	Filters	Results
1st search :<br><br>PubMed	("Sex"[Mesh] OR "Gender Role"[Mesh] OR "Gender [Mesh]" OR "Gender Identity"[Mesh]) AND ("Rectal Neoplasms"[Mesh] OR "Colorectal Neoplasms"[Mesh] OR "Colonic Neoplasms"[Mesh])	Results by years :<br><br>2000 to 2022	25 texts
2nd search :<br><br>PubMed	(((("Sex"[Title] OR "Gender Role"[Title] OR "Gender "[Title] OR "Gender Identity"[Title]))) AND ((("Colorectal cancer"[Title] OR "Colon Cancer"[Title] OR "Rectal Cancer"[Title])))) AND ("Incidence"[Title])	Results by years :<br><br>2000 to 2022	21 texts
Google scholar	allintitle: (sex OR gender) AND (rectal cancer OR colorectal cancer OR colon cancer)	Results by years :<br><br>2000 to 2022<br><br>(Without quotes)	37 texts
