# Supplementary material for: The Influence of Sex and/or Gender on the Occurrence of Colorectal Cancer in the General Population in Developed Countries: A Scoping Review
Source: Int J Public Health. 2024 Apr 10;69:1606736. doi: 10.3389/ijph.2024.1606736 (PMC11039791; doi:10.3389/ijph.2024.1606736)
Supplement: Supplementary file 2 [file Table2.pdf]

## Table S2: Characteristics of the selected studies (Toulouse, France. 2023)

| Authors | Publication year | Journal | Title | Country of origin | Data | Study population | Inclusion period | Sample size | Age range |
|---|---|---|---|---|---|---|---|---|---|
| **Abotchie et al** | 2011 | Europe PMC | Gender differences in colorectal cancer incidence in the United States, 1975-2006. | United States | Surveillance, Epidemiology and End Results (SEER) registries: San Francisco-Oakland, Connecticut, Detroit, Hawaï, Lowa, New Mexico, Seattle, Utah, Atlanta | Patients diagnosed with malignant CRC who resided in one of nine SEER regions of the United States. | 1975 to 2006 | 373 956 patients with invasive tumors | ≥ 40 years |
| **Cheng Whu et al.** | 2001 | ACS JOURNALS | Subsite-specific incidence rate and stage of disease in colorectal cancer by race, gender, and age group in the United States, 1992–1997 | United States | The North American Association of Central Cancer Registries (NAACCR) including 28 population-based central cancer registries included 11 registries in the SEER and 17 NPCR registries | Patients diagnosed with primary colorectal cancers which were miscropicall confirmed | 1992 to 1997 | 344775 participants registered in one of the 28 registers | 30 years to 85 and over |
| **Cook et al.** | 2009 | Cancer Epidemiol Biomarkers Prevention | Sex disparities in cancer incidence by period an age | United States | Data were extracted from the November 2007 submission of the SEER-9 registries database. | Patients diagnosed with malignant tumor who resided in one of nine Surveillance, Epidemiology and End Results (SEER) regions of the United States | periods 1975-2004 (75-84, 85 - 94, 95 - 04, 75 - 04) | | all ages |

| | | | | | | | | | |
|---|---|---|---|---|---|---|---|---|---|
| **Hoffmeister et al.** | 2010 | clinical gastroenterology and hepatology | Male Sex and Smoking Have a Larger Impact on the Prevalence of Colorectal Neoplasia Than Family History of Colorectal Cancer | Allemagne (Sarre (Saarland) | The data ascertained are baseline data of a statewide cohort study initiated in 2005. Patients were asked to fill out a standardized questionnaire on potential risk factors of CRC and to return the completed questionnaire to the study center by the appointed date of colonoscopy. Screening colonoscopy reports and histology reports were requested and transferred into a standardized form by double entry of 2 independent trained investigators who were blinded with respect to questionnaire data. | Population underwent screening colonoscopy for the first time within the nationwide colonoscopy screening program. Population with a fairly low level of education and more than two thirds overweight or obese | 2005 to 2007 | N=3349 | mean age was 63.5 years (included people who were 55 years or old) |
| **Murphy et al.** | 2011 | International journal of cancer | Sex Disparities in Colorectal Cancer Incidence by Anatomic Subsite, Race and Age | United States | The National Cancer Institute's Surveillance, Epidemiology, and End Results (SEER) program for cases diagnosed among residents of 13 registries during 1992–2006 | SEER registry patients diagnosed with a malignant tumor of the colon or rectum during 1992-2006. | 1992 to 2006 | NR | all ages |

| | | | | | | | | | |
|---|---|---|---|---|---|---|---|---|---|
| **Petrick et al.** | 2021 | FRONTIERS | Racial Disparities and Sex Differences in Early- and Late-Onset Colorectal Cancer Incidence | United States | Data from US Cancer Statistics, which includes data from the Centers for Disease Control and Prevention's National Program of Cancer Registries (NPCR) and the National Cancer Institute's SEER Program, spanning the years 2001 through 2018 (all available years) | Patients diagnosed with primary colorectal cancers. Characteristics of the US population because the US Cancer Statistics database covers 99% of the US population and therefore allows for comprehensive exploration of racial/ethnic and gender disparities | 2001 to 2018 | 2 585 621 CRC cases | 20-49 years / 50-74 years |
| **White et al.** | 2018 | BMC CANCER | A review of sex-related differences in colorectal cancer incidence, screening uptake, routes to diagnosis, cancer stage and survival in the UK | United Kingdom | The data used for the review included a cross-sectional study of colorectal cancer, compiling available national data for the UK (Wales, England and Northern Ireland). Data on incidence rates by age (2012-2014) and anatomical site (2010-2012) were taken from various publicly available datasets. | General population of the United Kingdom: a cross-sectional review of national data | 2006 to 2014 | 41 599 cases in 2010-2012 | all ages |
