# Supplementary figures and images for: The Influence of Sex and/or Gender on the Occurrence of Colorectal Cancer in the General Population in Developed Countries: A Scoping Review
Source: Int J Public Health. 2024 Apr 10;69:1606736. doi: 10.3389/ijph.2024.1606736 (PMC11039791; doi:10.3389/ijph.2024.1606736)

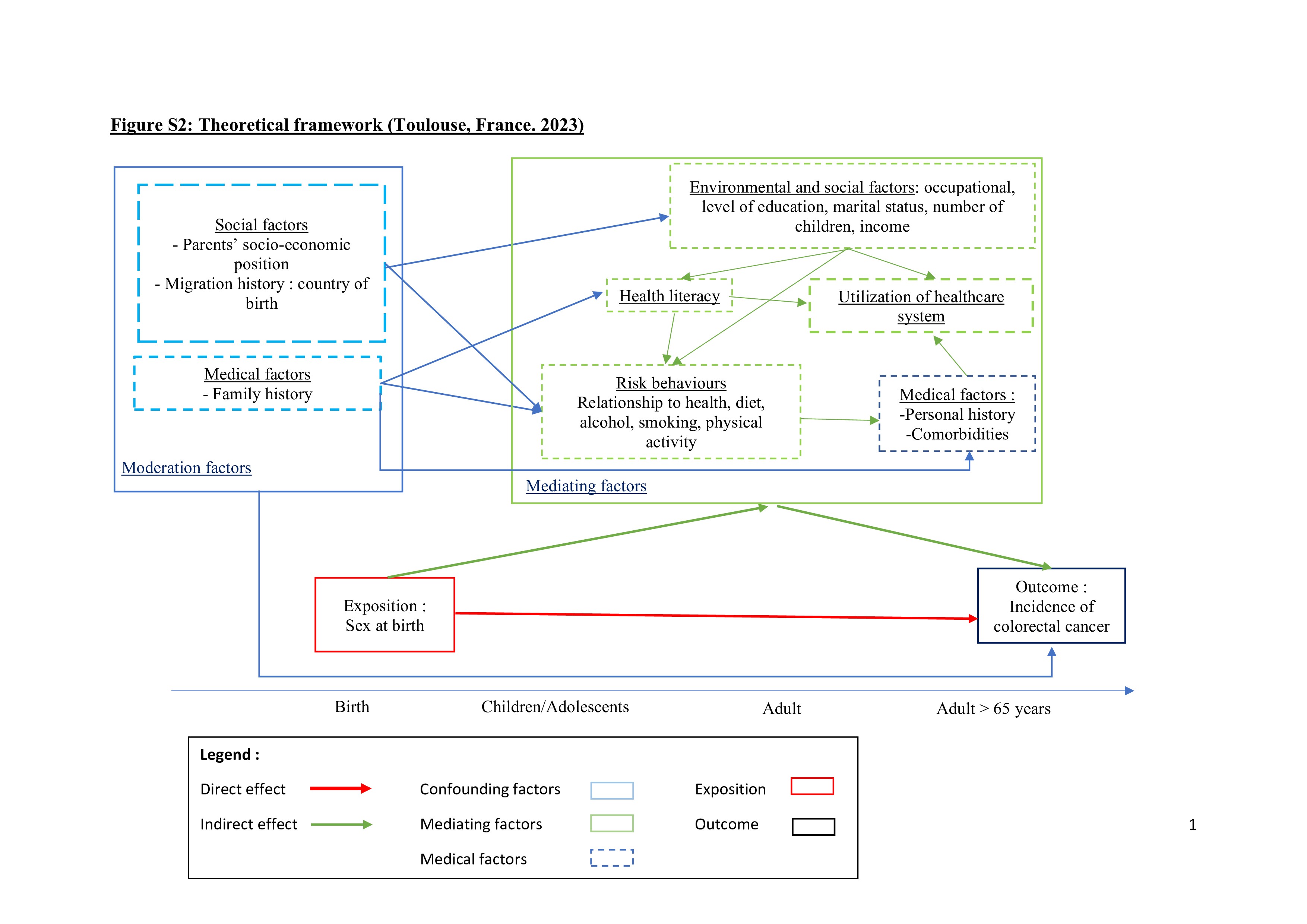

Supplement: Supplementary file 3 [file Image1.jpeg]
