# Supplementary material for: The Influence of Sex and/or Gender on the Occurrence of Colorectal Cancer in the General Population in Developed Countries: A Scoping Review
Source: Int J Public Health. 2024 Apr 10;69:1606736. doi: 10.3389/ijph.2024.1606736 (PMC11039791; doi:10.3389/ijph.2024.1606736)
Supplement: Supplementary file 4 [file Table3.pdf]

Table S3: Use of sex and/or gender in the articles and mechanism of gender (Toulouse, France. 2023)

Authors	Use terms to talk about their exposure[1]	Definition explicate of Sex/Gender?	How do they define the variable Sex/Gender?	Effect of sex/gender on CRC	Mechanism of gender
**Abotchie and al.**	sex: 0<br>gender: 29	NO	*Introduction:* "Although gender is often acknowledged as an independent influence on CRC incidence […] few have examined how these factors differ between men and women and how differences may vary over time. Gender typically is controlled for in analysis. As a result, our understanding of most influences on health-related outcomes is based on effects independent of gender"<br>*Objective*: […] "(2) examining the changes in gender differences over time by age, race, and tumor stage, and (3) investigating gender differences by geographic areas. The detailed examination of these factors may enhance our understanding of potential causes of gender differences in CRC incidence."<br>*Methods*: their "primary independent variable […] was gender (male of female)"	**Main exposure**: **"Gender"**: "The incidence rate ratio between men and women was 1.38. The higher incidence rates in men than in women persisted in all age strata, tumor sites, tumor stages, and geographic area."	It would appear that the authors want to understand how risk factors differ between men and women and how this difference may impact the risk of colorectal cancer. The integration of geographical area and race which reflect socio-demographic indicators will allow a gendered dimension of sex. Consequently, with regard to their non-explicit gender hypothesis, the socio-demographic factors on which the authors will adjust the effect of gender on the incidence of colorectal cancer allows the integration of the gender mechanism.
**Cheng Whu and al.**	sex: 0<br>gender: 14	NO	*In introduction*: "The variations of the subsite specific incidence rate by age, gender, and race may be related not only to their distinctions in exposure to these factors but also to strategies of screening for colorectal cancer because the likelihood of early detection by screening procedures is largely dependent on the anatomic subsite where colorectal cancer develops."<br>*Objective*: "We examined the subsite specific cancer incidence rates and relation of stage of	"Cancer incidence rates were significantly higher in males than in females in all anatomic subsites. The male-to-female rate ratios were progressively higher from cecum to rectum regardless of race, and all were statistically significant."	The authors state that the incidence of CRC is influenced by exposure to certain risk factors and screening strategies. however, the authors did not include diagnostic pathways in their analyses. However, in view of their assertions of exposure to different factors according to the sexes and the exclusive use of the term "gender" to describe their

			disease with anatomic subsite by race, gender, and age group by using the largest aggregation of cancer incidence data amassed to date to our knowledge from population-based registries in the United States. The analysis may provide insight into demographic patterns and their affiliation to colorectal cancer risk and stage of disease at diagnosis at particular anatomic subsites."		exposure variable, the authors seem to be using gender mechanisms. By adjusting for race, the authors no doubt want to introduce a social factor to allow the integration of gender mechanisms in their analyses.
**Cook and al.**	sex: 19<br>gender: 0	YES	*Introduction*: "Sex, that is, being male or female, is an important basic human variable that should be considered when designing and analyzing studies in all areas and at all levels of biomedical and health-related research. Differences in health and illness are influenced by individual genetic and physiological constitutions, as well as by an individual's interaction with environmental and experimental factors. The incidence and severity of diseases vary between the sexes and may be related to differences in exposures, routes of entry and the processing of a foreign agent, and cellular responses."<br>*Objective*: "This study uses data from the Surveillance, Epidemiology and End Results (SEER) cancer registry program to analyze age-adjusted male-to-female incidence rate ratios (IRR) and sex-specific incidence rates by cancer site, histology, year of diagnosis, and age of diagnosis. It is hoped that this exercise will be hypothesis-generating with regards to sex differences in the etiopathogenesis of cancer."	**main exposure: "sex":** Higher incidence rate in men than in women. The male-to-female Incidence rate ratios (IRR) (95%CI) for 1975 - 2004 years, was 1,37 (1,37-1,38)	According them, sex exposes to different risk factors of cancer, like carcinogenic exposure, biology and risk behavior.<br>Despite the repeated use of the word "sex" the authors do highlight gender mechanism.
**Hoffmeister and al.**	sex: 2<br>gender: 7	NO	*Objective: "*We conducted a population-based study with 3349 first-time participants of colonoscopy within a real screening setting in Germany and aimed to investigate prevalences, prevalence ratios (PRs), and population attributable fractions (PAFs) of colorectal polyps associated with smoking, gender, and family history of CRC."	"PRs of all types of polyps were consistently higher in men compared with women"<br>"In the studied population, PAFs of non-advanced and advanced neoplasia were highest for male gender (23% and 23%, respectively)	The authors analysed the risk of CRC through gender and associated behaviours (use of health care and smoking status), which shows the socio-cultural role of gender. The authors mainly use the word gender without an associated definition, and differentiate it from sex. They often

			Methods: "Apart from age at colonoscopy, gender, cigarette smoking status (never, former, current), and family history of CRC (any parent or sibling and any age of diagnosis), the following covariates were included in the model because they were differentially distributed between polyp-positive and polyp-free patients (P< .20): school education (<9, 10 –11, 12–13 years), body mass index, physical activity (quartiles of metabolic equivalents), alcohol consumption (no alcohol and quartiles of amount of ethanol in grams), daily consumption of red meat (yes/no), ever use of hormone replacement therapy (yes/no)."	followed by history of smoking (7% and 9%, respectively)". "the population fraction of non-advanced or advanced neoplasia attributable to a single risk factor was highest for male gender".	associate male and gender which implies behaviours and lifestyles specific to the male category.
**Murphy and al.**	sex: 9<br>gender: 0	NO	*Introduction:* "the male-to-female incidence rate ratio (MF IRR) increases progressively across the colon from the cecum to the rectum. This pattern is unexplained and likely a result of a combination of better awareness of screening in women, sex-specific exposure to risk factors, and protective effects of both endogenous and exogenous hormones"<br>*Objective*: "We investigated recent CRC incidence data according to anatomic subsite, race/ethnicity and age, focusing on the sex-specific patterns to broaden our understanding and reveal potential clues for etiologic research."	**main exposure: "sex": "**Male rates were higher than female rates at all subsites for all racial/ethnic groups, with the single exception of proximal CRC sites for American Indian/Alaska Native, where female rates were slightly higher than male"	From the outset, the authors express the hypothesis of a sex-linked gender mechanism by assuming gendered health-seeking behaviors but also more frequent exposure to risk factors according to gender. In view of their hypothesis, it is understandable that the inclusion of race in their analyses implies an approximation of racial and socio-economic factors which, when linked to sex, allows for a gender mechanism.
**Petrick and al.**	sex: 9<br>gender: 0	NO	*Introduction:* "Established risk factors for late-onset CRC, including obesity, physical inactivity, smoking, alcohol, and diet, have also been associated with EOCRC risk in some studies. However, known CRC risk factors do not fully explain the increasing rates of EOCRC, indicating there are likely undiscovered risk factors for EOCRC."<br>*Objective: "*In the present study, we examined US CRC rates from 2001-2018 by age, sex, race/ ethnicity, anatomic location, and histology. The	**main exposure: "sex":** "Sex differences were observed for both early- and late-onset CRC, with men having 16% higher rates of EOCRC and 44% higher rates of late-onset CRC."	The authors claim that social factors such as physical inactivity, obesity, diet, tobacco and/or alcohol consumption increase the risk of colorectal cancer. They assume that there are still risk factors to be discovered but which, obviously, would not be social. Moreover, the authors do not associate gender with these risk factors, except in discussion.

			trends of EOCRC by these factors may provide clues as to the evolving etiology of EOCRC." (early-onset colorectal cancer = EOCRC)"		Given their claims, even adjusting for race, the authors seem to use the sex assigned at birth as an exposure variable.
**White and al.**	sex: 20<br>gender: 4	NO	*Introduction*: "Colorectal cancer (CRC) is an illness strongly influenced by sex and gender, with mortality rates in males significantly higher than females." "CRC is a disease that has both biological sex differences and socio-cultural gender components."	**main exposure: "sex and gender"**: "There are small differences in the stage distribution of CRCs at diagnosis between males and females"<br>When there is an adjustment on the diagnostic pathways the effect of sex changes: despite an overall higher participation in the generalized screening program among women, men still represent the most cases of CRC (8.1% of male CRCs vs 5.1% of female CRCs), but when the diagnosis is done in an emergency, women are much more affected (27.6% female CRCs vs 22.1% male CRCs)	They distinguish sex and even if it is not explicit in the methodology and the analyses, by describing the health behaviors linked to the use of the healthcare system according to gender, we can see a gendered behavior.

[1]use of words sex and/or gender from the title, aim, methods and results of the text and abstract, to define their exposure variable SEX/GENDER (without discussion and conclusion)
