# Supplementary material for: The Influence of Sex and/or Gender on the Occurrence of Colorectal Cancer in the General Population in Developed Countries: A Scoping Review
Source: Int J Public Health. 2024 Apr 10;69:1606736. doi: 10.3389/ijph.2024.1606736 (PMC11039791; doi:10.3389/ijph.2024.1606736)
Supplement: Supplementary file 5 [file DataSheet1.pdf]

Appendix:

Table 1: Selection of articles on the title (Toulouse, France. 2023)

Authors	Title	Publication date	Selected on the title
**Abotchie P. and al.**	Gender Differences in Colorectal Cancer Incidence in the United States, 1975–2006	2012	**YES**
**Abrahamsson and al.**	Sex disparities in vitamin D status and the impact on systemic inflammation and survival in rectal cancer	2021	**YES**
**Adam S. and al.**	Cancer Treatment Delays in American Indians and Alaska Natives Enrolled in Medicare.	2017	NO
**Ameri and al.**	Different irradiation machines and their effects on testes' exposure levels and sex hormones profile in patients with rectal cancer	2010	NO
**Ameri and al.**	Testes doses and sex hormone changes using different radiotherapy machines to treat rectal cancer	2009	NO
**Anderson and al.**	Body Mass Index A Marker for Significant Colorectal Neoplasia in a Screening Population	2007	NO
**Arbeev K. and al.**	Difference between Male and Female Cancer Incidence Rates: How Can It Be Explained?	2006	**YES**
**Arslani and al.**	Influence of gender, ASA physical status, the location of a tumor and stage of the disease on the survival rate in patients with rectal cancer after surgery." Nigerian Journal of Clinical Practice, vol. 23, no. 11, Nov. 2020, p. 1514. Gale OneFile: Health and Medicine,	2022	NO
**Bakke and al.**	Sex Differences and Tumor Blood Flow from Dynamic Susceptibility Contrast MRI Are Associated with Treatment Response after Chemoradiation and Long-term Survival in Rectal Cancer	2020	NO
**Berger and al.**	Impact of sex, age, and ethnicity/race on the survival of patients with rectal cancer in the United States from 1988 to 2012	2016	NO
**Brooks and al.**	The Black Panther, Masculinity Barriers to Medical Care, and Colorectal Cancer Screening Intention Among Unscreened American Indian/Alaska Native, Black, and White Men.	2022 Apr	NO
**Burnett-Hartman and al.**	Differences in epidemiologic risk factors for colorectal adenomas and serrated polyps by lesion severity and anatomical site	2013 Apr	**YES**

**Campbell and al.**	Body mass index and risk of colon and rectal cancer stratified by tumor location and sex Author links open overlay panel	2004	**YES**
**Carethers and al.**	How Masculinity Affects Colorectal Cancer Screening in African-American Men	2022 Feb	NO
**Chao and al.**	Meat consumption and risk of colorectal cancer	2005	NO
**Charkhchi and al.**	Modifiers of Cancer Screening Prevention Among Sexual and Gender Minorities in the Behavioral Risk Factor Surveillance System.	2019 Apr	NO
**Christy M. and al.**	Integrating men's health and masculinity theories to explain colorectal cancer screening behavior	2014 Jan	NO
**Clark MA. and al.**	Measuring sexual orientation and gender expression among middle-aged and older women in a cancer screening study	2005 Summer	NO
**Conti L. and al.**	Revisiting the impact of lifestyle on colorectal cancer risk in a gender perspective.	2010	**YES**
**Cook and al.**	Sex Disparities in Cancer Incidence by Period and Age	2009	**YES**
**Cross and al.**	A large prospective study of meat consumption and colorectal cancer risk: an investigation of potential mechanisms underlying this association	2010	NO
**Diefenhardt and al.**	Association of Sex With Toxic Effects, Treatment Adherence, and Oncologic Outcomes in the CAO/ARO/AIO-94 and CAO/ARO/AIO-04 Phase 3 Randomized Clinical Trials of Rectal Cancer	2019	NO
**Earl V. and al.**	Barriers and Facilitators to Colorectal Cancer Screening in African-American Men	2022 Feb	NO
**Emslie C. and al.**	Getting through' not 'going under': a qualitative study of gender and spousal support after diagnosis with colorectal cancer.	2009 Mar	NO
**Fietkau and al.**	Rectal cancer delivery of radiotherapy in adequate time and with adequate dose is influenced by treatment center, treatment schedule, and gender and is prognostic parameter for local control: Results of study CAO/ARO/AIO-94	2007	NO
**Getrich CM. and al.**	Expressions of machismo in colorectal cancer screening among New Mexico Hispanic subpopulations.	2012 Apr	NO
**Gao and al.**	Race and Gender Predilection for Spectroscopic Rectal Microvascular Markers in Colonic Field Carcinogenesis Detection: Implications for Colorectal Cancer Screening	2015	NO
**Gasinska and al.**	Gender-Related Differences in Repopulation and Early Tumor Response to Preoperative Radiotherapy in Rectal Cancer Patients	2011	NO
**Gasinska and al.**	Gender-Related Differences in Pathological and Clinical Tumor Response Based on Immunohistochemical Proteins Expression in Rectal Cancer Patients Treated with Short Course of Preoperative Radiotherapy	2014	NO

**Gasinska and al.**	Gender-related prognostic significance of clinical and biological tumor features in rectal cancer patients receiving short-course preoperative radiotherapy	2017	NO
**Gasinska and al.**	Gender-related significance of time interval between radiotherapy and surgery in hypofractionated preoperative radiotherapy for rectal cancer patients' survival	2016	NO
**Glover and al.**	Epidemiology of Colorectal Cancer in Average Risk Adults 20-39 Years of Age: A Population-Based National Study	2019	NO
**Hansen and al.**	Possible better long-term survival in left versus right-sided colon cancer - a systematic review	2012	NO
**Haziman AA. and al.**	A novel role for estrogen-induced signaling in the colorectal cancer gender bias.	2019 May	NO
**Heath A. K. and al.**	Circulating 25-Hydroxyvitamin D Concentration and Risk of Breast, Prostate, and Colorectal Cancers: The Melbourne Collaborative Cohort Study	2019	NO
**Hendifar A. and al.**	Gender Disparities in Metastatic Colorectal Cancer Survival	2009	NO
**Hian Koo J. and al.**	Sex differences in epidemiological, clinical and pathological characteristics of colorectal cancer	2009	**YES**
**Hoffmeister M. and al.**	Male Sex and Smoking Have a Larger Impact on the Prevalence of Colorectal Neoplasia Than Family History of Colorectal Cancer	2010	**YES**
**Jacobs and al.**	Diet, gender, and colorectal neoplasia	2007	**YES**
**Katzenstein and al.**	Gender-specific differences of the early postoperative and oncosurgical long-term outcome in rectal cancer-data obtained in a prospective multicenter observational study].	2018	NO
**Kim Sung-Eun and al.**	Sex- and gender-specific disparities in colorectal cancer risk	2015	**YES**
**Lamiae and al.**	Influence of Gender on Clinical, Pathological and Prognostic Characteristics of Rectal Cancer in Morocco: A Single Institution Experience	2014	NO
**Lim JW. and al.**	Gender and Role Differences in Couples' Communication During Cancer Survivorship	2015 May-Jun	NO
**McCaughan and al.**	Exploring and comparing the experience and coping behaviour of men and women with colorectal cancer at diagnosis and during surgery.	2011 Jul	NO
**Murphy G. and.**	Sex Disparities in Colorectal Cancer Incidence by Anatomic Subsite, Race and Age	2011	**YES**
**Nguyen S. and.**	Gender as a risk factor for advanced neoplasia and colorectal cancer: a systematic review and meta-analysis	2009	**YES**
**Okafor and al.**	Gender and Socioeconomic Disparities in the Locoregional Staging of Rectal Cancer Impact Treatment Patterns and Survival	2015	NO

**Ong and al.**	Regional and national guideline recommendations for digital ano-rectal examination as a means for anal cancer screening in HIV positive men who have sex with men: a systematic review	2014	NO
**Petrick JL and al.**	Racial Disparities and Sex Differences in Early- and Late-Onset Colorectal Cancer Incidence, 2001–2018	2021	**YES**
**Peila and al.**	Sex hormones, SHBG and risk of colon and rectal cancer among men and women in the UK Biobank	2017	NO
**Pocard and al.**	A prospective study of sexual and urinary function before and after total mesorectal excision with autonomic nerve preservation for rectal cancer	2002 Apr	NO
**Rennoldson and al.**	A discursive psychology analysis of emotional support for men with colorectal cancer.	2013 Nov	NO
**Rezende Dázio E.M and al.**	The meaning of being a man with intestinal stoma due to colorectal cancer: an anthropological approach to masculinities.	2009 Sep-Oct	NO
**Rogers and al.**	Study protocol for developing #CuttingCRC: a barbershop-based trial on masculinity barriers to care and colorectal cancer screening uptake among African-American men using an exploratory sequential mixed-methods design.	2019 Jul	NO
**Rogers and al.**	Masculinity Barriers to Ever Completing Colorectal Cancer Screening among American Indian/Alaska Native, Black, and White Men (Ages 45-75).	2022 Mar	NO
**Rundle and al.**	Colonoscopic Screening in Average-Risk Individuals Ages 40 to 49 vs 50 to 59 Years	2008	NO
**Sarasqueta and al.**	Gender differences in stage at diagnosis and preoperative radiotherapy in patients with rectal cancer.	2020	**YES**
**Schoenfeld and al.**	Colonoscopic Screening of Average-Risk Women for Colorectal Neoplasia	2005	NO
**Seike K. and al.**	Gender differences in pelvic anatomy and effects on rectal cancer surgery.	2009	NO
**Shinji and al.**	Male sex and history of ischemic heart disease are major risk factors for anastomotic leakage after laparoscopic anterior resection in patients with rectal cancer.	2018	NO
**Spain S. and al.**	Colorectal cancer risk is not associated with increased levels of homozygosity in a population from the United Kingdom.	2009 Sep	NO
**b and al.**	Fertility Preservation Discussions Between Young Adult Rectal Cancer Survivors and Their Providers: Sex-Specific Prevalence and Correlates	2022	NO

**Tepper and al.**	Adjuvant Therapy in Rectal Cancer: Analysis of Stage, Sex, and Local Control—Final Report of Intergroup 0114 | Journal of Clinical Oncology (ascopubs.org)	2016	NO
**Thompson and al.**	I can't get my husband to go and have a colonoscopy: gender and screening for colorectal cancer.	2012 May	NO
**Thong S Y. and al.**	Age at Diagnosis and Sex Are Associated With Long-term Deficits in Disease-Specific Health-Related Quality of Life of Survivors of Colon and Rectal Cancer: A Population-Based Study	2019	NO
**Tsiouris A. and al.**	What is the Image of the "Typical Cancer Patient"? The View of Physicians.	2021 Mar-Apr	NO
**Warren and al.**	A novel interaction of genotype, gender, and adjuvant treatment in survival after resection of stage III colon cancer: Results of CALGB 89803.	2012	NO
**White A. and al.**	A review of sex-related differences in colorectal cancer incidence, screening uptake, routes to diagnosis, cancer stage and survival in the UK	2018	**YES**
**Wichmann M. W. and al.**	Gender differences in long-term survival of patients with colorectal cancer, British Journal of Surgery,	2001	NO
**Winterich JA. and al.**	Masculinity and the body: how African American and White men experience cancer screening exams involving the rectum.	2009 Dec	NO
**Worrall F. and al.**	Priorities of Unmet Needs for Those Affected by Colorectal Cancer: Considerations From a Series of Nominal Group Technique Sessions.	2021 Feb	NO
**WU XC. and al.**	Subsite-specific incidence rate and stage of disease in colorectal cancer by race, gender, and age group in the United States, 1992–1997†	2001	**YES**
**Wyrwicz and al.**	Effect of sex on treatment outcomes in rectal cancer patients after neoadjuvant radiotherapy or chemoradiation: A combined analysis of Polish-1 and Polish-2 studies	2017	NO
**Zutshi and al.**	Gender differences in mortality, quality of life and function after restorative procedures for rectal cancer	2012	NO
